# Surveillance and Phylogenetic Characterisation of Avian Influenza Viruses Isolated from Wild Waterfowl in Zambia in 2015, 2020, and 2021

**DOI:** 10.1155/2023/4606850

**Published:** 2023-03-01

**Authors:** Annie Kalonda, Ngonda Saasa, Masahiro Kajihara, Naganori Nao, Ladislav Moonga, Joseph Ndebe, Akina Mori-Kajihara, Andrew Nalishuwa Mukubesa, Mulemba Samutela, Samuel Munjita, Yoshihiro Sakoda, Hirofumi Sawa, Ayato Takada, Edgar Simulundu

**Affiliations:** ^1^Department of Biomedical Sciences, School of Health Sciences, University of Zambia, Lusaka 10101, Zambia; ^2^Department of Disease Control, School of Veterinary Medicine, University of Zambia, Lusaka 10101, Zambia; ^3^Africa Centre of Excellence for Infectious Diseases of Humans and Animals, School of Veterinary Medicine, University of Zambia, Lusaka 10101, Zambia; ^4^Division of International Research Promotion, International Institute for Zoonosis Control, Hokkaido University, N20 W10, Kita-ku, Sapporo 001-0020, Japan; ^5^Hokudai Center for Zoonosis Control in Zambia, School of Veterinary Medicine, University of Zambia, Lusaka 10101, Zambia; ^6^One Health Research Center, Hokkaido University, N18 W9, Kita-ku, Sapporo 001-0020, Japan; ^7^Department of Paraclinical Studies, School of Veterinary Medicine, University of Zambia, Lusaka 10101, Zambia; ^8^Division of Global Epidemiology, International Institute for Zoonosis Control, Hokkaido University, N 20 W10, Kita-ku, Sapporo 001-0020, Japan; ^9^International Collaboration Unit, International Institute for Zoonosis Control, Hokkaido University, N20 W10, Kita-ku, Sapporo 001-0020, Japan; ^10^Laboratory of Microbiology, Department of Disease Control, Faculty of Veterinary Medicine, Hokkaido University, Sapporo, Hokkaido 060-0818, Japan; ^11^Hokkaido University, Institute for Vaccine Research and Development (HU-IVReD), N21 W11, Kita-ku, Sapporo 001-0020, Japan; ^12^Division of Molecular Pathobiology, International Institute for Zoonosis Control, Hokkaido University, N20 W10, Kita-ku, Sapporo 001-0020, Japan; ^13^Global Virus Network, 725 W Lombard Street, Baltimore, MD 21201, USA; ^14^Macha Research Trust, Choma 20100, Zambia

## Abstract

In recent years, the southern African region has experienced repeated incursions of highly pathogenic avian influenza viruses (HPAIVs), with wild migratory birds being implicated in the spread. To understand the profile of avian influenza viruses (AIVs) circulating in Zambia, we surveyed wild waterfowl for AIVs and phylogenetically characterised the isolates detected in 2015, 2020, and 2021. A total of 2,851 faecal samples of wild waterfowl were collected from Lochinvar National Park in the Southern Province of Zambia. During the study period, 85 (3.0%) low pathogenicity AIVs belonging to various subtypes were isolated, with H2N9, H8N4, and H10N8 being reported for the first time in avian species in Africa. The majority of the isolates were detected from glossy ibis (order *Pelecaniformes*) making it the first report of AIV from these birds in Zambia. Phylogenetic analysis of all eight gene segments of the 30 full genomes obtained in this study revealed that all the isolates belonged to the Eurasian lineage with their closest relatives being viruses isolated from wild and/or domestic birds in Bangladesh, Belgium, Egypt, Georgia, Mongolia, the Netherlands, and South Africa. Additionally, the Zambian viruses were grouped into distinct clusters based on the year of isolation. While no notifiable AIVs of the H5 or H7 subtypes were detected in wild birds in Zambia, viral internal protein genes of some viruses were closely related to H7 low pathogenicity AIVs. This study shows that periodically, a considerable diversity of AIV subtypes are introduced into the Zambian ecosystem by wild migratory waterfowl. The findings highlight the importance of continuous surveillance and monitoring of AIVs in wild waterfowl, including birds traditionally not considered to be major AIV reservoirs, for a better understanding of the eco-epidemiology and evolutionary dynamics of AIVs in Africa.

## 1. Introduction

Avian influenza viruses (AIVs) are of global concern, with some subtypes and strains posing a constant threat to the poultry industry [1]. They are segmented, negative-sense RNA viruses belonging to the *Orthomyxoviridae* family, genus *Alpha influenza virus*. Wild waterfowl are the known natural reservoir of AIVs and play an important role in the evolution and spread of these viruses [2, 3]. Wild waterfowl of the orders *Anseriformes* and *Charadriiformes* are thought to be the most common reservoirs of diverse AIV subtypes, including 16 distinct haemagglutinin (HA) (H1–H16) and nine neuraminidase (NA) (N1–N9) [4]. Wild waterfowl can carry AIVs from one area to another and present a formidable risk to susceptible host species along their migratory flyway [5]. Therefore, wild waterfowl are of primary interest in AIV surveillance efforts, especially in places where birds of various geographical origins congregate at high densities, such as at stopover sites within migratory bottlenecks, creating so-called transmission “hotspots” [6].

AIVs exist in two forms: low and high pathogenicity viruses based on their pathotypes in chickens. Low pathogenicity AIVs (LPAIVs) circulating widely in waterfowl generally cause asymptomatic infections in these birds and are primarily shed in faeces [5]. In addition, LPAIVs are known to cause mild to no symptoms in chickens due to their restricted replication in the respiratory and/or intestinal tracts [7]. By contrast, high pathogenicity avian influenza viruses (HPAIVs) cause systemic infections and high mortality of up to 100% [8]. Particularly, the Gs/Gd-lineage H5N1 HPAIV that was first reported in Hong Kong in 1997 and H5Nx HPAIVs whose HA have the same origin as the H5N1 virus have caused a large number of outbreaks in poultry and wild birds across the globe [8, 9]. So far, HPAIVs have been restricted to subtypes H5 and H7, although not all viruses of these subtypes cause high pathogenicity avian influenza (HPAI) [4, 9, 10]. However, H5 and H7 LPAIVs may evolve spontaneously into HPAIVs, especially upon introduction into birds of the order *Galliformes* [5, 11–14].

In Africa, the first report of HPAI outbreak was recorded in 1961 during which approximately 1,300 common terns died in South Africa [15, 16]. Since then, no reports of HPAI outbreaks were recorded in Africa until 2004 when an outbreak caused by an H5N2 HPAIV was reported in South African ostriches [17]. In 2006, the Gs/Gd-lineage H5N1 HPAIV infection of commercial poultry was first confirmed on the continent on farms in Kaduna state in northern Nigeria [18, 19]. The virus spread rapidly to various African countries and resulted in losses of unprecedented proportions to the poultry industry, impacting national economies and international trade of live poultry and poultry products in the affected countries [20]. Moreover, the virus was thought to have been introduced through routes that coincided with the flight pathways of migratory birds [19], highlighting the importance of migratory birds in AIV dispersal including that of HPAIVs. Since the first outbreak of the Gs/Gd-lineage H5N1 HPAIV infection was reported in 2006 in Africa, two more waves of H5Nx HPAIVs have been confirmed including the H5N1 viruses from clade 2.3.2.1c isolated in 2015 and the 2016 clade 2.3.4.4b H5N8 viruses which emerged in western Africa and spread to southern, central, and eastern Africa [20, 21]. In southern Africa, clade 2.3.4.4b H5N8 viruses reached the Democratic Republic of the Congo (DR Congo), Zimbabwe, and South Africa in 2017 [22, 23]. In February 2019, new cases were reported in Namibia [24]. Furthermore, the HPAI outbreak caused by the H5N1 clade 2.3.4.4b virus was recently reported in Botswana [25]. Remarkably, despite several reports of HPAI outbreaks in neighbouring countries, there has been no report of HPAIVs in Zambia, possibly due to nondominance of the strain or due to a break in surveillance of AIVs in the country.

Apart from the havoc caused by HPAIVs, H9N2 LPAIV in poultry has gained attention because of the serious repercussions on animal health, public health, and the trade of live poultry or poultry products. Since their detection in China in 1992 [26], H9N2 LPAIVs have been extensively circulating in North African countries since the early 2000s [27, 28]. From 2017 to date, H9N2 viruses have been detected in several sub-Saharan African countries: Ghana, Burkina Faso, Uganda, Kenya, and Senegal, where a human case was recently reported [26, 29–31]. Surveillance studies have also been conducted in wild waterfowl in Africa which led to the detection of diverse subtypes of LPAIVs [32–35]. Our AIV surveillance in Zambia in 2006 and 2008–2009 resulted in the isolation of 13 viruses of distinct subtypes (H3N6, H3N8, H4N6, H6N2, H9N1, and H11N9) from wild waterfowl [36, 37]. However, compared to surveillance activities in other regions of the world, AIV surveillance in wild birds in Africa, especially southern Africa, is patchy and limited in geographical coverage [38]. Nevertheless, surveillance for AIVs in wild birds should not be underestimated as these viruses have contributed some gene segments to virus strains that have caused human influenza pandemics [39–41] and have the potential to cause future pandemics. Moreover, from Africa, only a few complete AIV sequences have been deposited in public databases suggesting a gap in the surveillance activities on the continent as a whole. Here, we phylogenetically characterised AIVs obtained from wild waterfowl in Zambia to better understand the profile of circulating viruses in these hosts.

## 2. Materials and Methods

### 2.1. Study Area and Sample Collection

A total of 2,851 fresh faecal samples from various wild waterfowl were collected in 2015, 2020, and 2021 from Lochinvar National Park (LNP) in the Southern Province of Zambia. The LNP (15°51′S 27°13′E) is home to over 420 bird species and more than 30,000 endemic Kafue lechwe (*Kobus leche kafuensis*) (Kafue lechwe are a species of antelopes found in the Kafue flats in Zambia) [42]. The LNP is important for continued AIV surveillance as it receives migratory birds, and various AIV subtypes have been previously detected in faecal samples of birds found in this park [36, 37]. Sample collection was carried out once every month from January–February and May–December in 2015, September and October in 2020, and October–December in 2021. Approximately 200 samples were collected each month except for some months in the rainy season (March–April 2015 and January–April 2020–2021) when the wetland was inaccessible due to extreme flooding. Furthermore, sampling was not carried out in the other dry months of 2020 and 2021 due to public health restrictions on movement during the coronavirus disease 2019 (COVID-19) pandemic. Our sampling strategy was to collect samples from sites where waterfowl were physically seen congregating for easier morphological identification of the birds by the trained ornithologist from the Department of National Parks and Wildlife and collecting well-separated fresh faecal samples. A sample was collected from each fresh faecal material in the field. Samples were kept at 4°C and transported to the University of Zambia, School of Veterinary Medicine laboratories for further analysis within 24 hours of being collected.

### 2.2. Virus Isolation and Subtyping

Faecal samples were processed according to the previously described protocol [36, 43]. Briefly, faecal samples were suspended in phosphate-buffered saline (PBS) (pH 7.4) supplemented with antimicrobials to prepare a 20% homogenate. The antimicrobial supplements included penicillin G (2 × 10^6^ U/litre), streptomycin (200 mg/litre), gentamycin (250 mg/litre), and nystatin (0.5 × 10^6^ U/litre) (Meiji Seika Pharma Co., Ltd, Tokyo, Japan). The faecal homogenates were centrifuged at 2000 ×g for 10 min at 4°C. Then, 0.5 ml of the supernatant was inoculated into the allantoic cavity of 9 to 11-day-old embryonated chicken eggs (two eggs per homogenate sample). The eggs were incubated at 37°C for 48 hours and then chilled overnight at 4°C. The amino-allantoic fluids (AAFs) collected from the eggs were screened by a haemagglutination (HA) test with 0.5% chicken red blood cells. The AAF samples that did not show HA activity were passaged to a second egg inoculation followed by an HA test. Samples that were HA-negative on the second passage were considered negative. Haemagglutination-inhibition (HI) and neuraminidase-inhibition (NI) assays were used to determine the HA and NA subtypes by using a panel of hyperimmune antisera against H1 to H16 and N1 to N9 subtypes, respectively, according to the standard protocol [43].

### 2.3. RNA Extraction and Whole Genome Sequencing

RNA was extracted from the AAF using the QIAamp Viral RNA Mini Kit (Qiagen, Hilden, Germany) according to the manufacturer's protocol. The extracted RNA was subjected to next-generation sequencing (NGS) using the Illumina MiSeq System (Illumina, San Diego, CA, USA). Libraries were prepared using KAPA RNA Hyper Prep Kit (Illumina, Inc., San Diego, CA, USA) and KAPA Dual-Indexed Adapter Kit (Roche, Basel, Switzerland). Libraries were then purified with Agencourt®AMPure®XP beads (Beckman Coulter, Brea, CA, USA). The library quantity and quality were verified using Agilent High Sensitivity DNA Kit on an Agilent 2100 Bioanalyzer (Agilent Technologies Inc., Santa Clara, CA, USA). For sequencing, the pooled libraries were diluted to a final concentration of 10 pM, followed by the addition of a 3% PhiX control library (Illumina, San Diego, CA, USA). The prepared libraries were sequenced on a MiSeq by using MiSeq Reagent kit v3 (600 cycles) (Illumina, San Diego, CA, USA) with 2 × 300 bp paired-end read length. Sequence reads were mapped to reference sequences of AIV gene segments, and the consensus sequences were rebuilt until all mismatches were solved using the CLC Genomic Workbench, version 22.0 (CLC bio, Aarhus, Denmark). The nucleotide sequences obtained in this study were submitted to GenBank under accession numbers OQ120633 to OQ120872.

### 2.4. Phylogenetic and Molecular Analysis

Nucleotide similarity searches were performed on the National Centre for Biotechnology Information (NCBI) website with the Basic Local Alignment Search Tool (BLAST) (https://blast.ncbi.nlm.nih.gov/Blast.cgi). For phylogenetic analysis, reference sequences were obtained from the NCBI database and included viruses that showed high sequence identity to our isolates, representative viruses from Africa, and viruses previously isolated in Zambia. Multiple sequence alignment was performed using the Multiple Alignment with Fast Fourier Transformation (MAFFT) (https: https://mafft.cbrc.jp/alignment/software/) (Accessed on 1^st^ December 2022) according to default parameters [44], and the aligned sequences were manually edited and trimmed using Geneious Prime® v2022.2.2. Maximum likelihood trees of all the eight full gene segments (PB2, PB1, PA, HA, NP, NA, M, and NS) of all the sequenced isolates with the exception of the HA gene for H2 isolates were generated using Molecular Evolutionary Genetics Analysis version X (MEGA X) [45, 46] applying the Tamura-Nei model [47] and 1,000 replicates of bootstrap. For the H2 isolates, the partial HA genes were used to allow for the inclusion of two sequences from Africa (Réunion Island). The software, GENETYX Version 12.0 (Genetyx Co., Tokyo, Japan), was used to assess the HA cleavage site of all the Zambian isolates.

## 3. Results

### 3.1. Surveillance of AIVs in Wild Waterfowl

In 2015, 2020, and 2021, a total of 2,851 faecal samples of wild waterfowl were collected from LNP in the Southern Province of Zambia. A total of 85 samples obtained from ducks, geese, and ibises were positive for AIV with a positivity rate of 3.0% by virus isolation as shown in Table 1. The positivity rate differed among bird species, seasons, and sampling years. Among the bird species, the positivity rate was highest in ibis birds (23.8%), followed by ducks (0.8%), and geese (0.2%). No virus was detected in great white pelicans and white egrets. High positivity rates were observed in the wet season (5.7%) and the 2015 (4.1%) sampling period (Table 1).

Based on the HI and NI assay results, 10 HA/NA subtype combinations were detected, namely, H2N9, H3N8, H4N6, H8N9, H10N7, H10N8, H10N9, H11N8, H11N9, and H13N6 and three HA subtypes, H1, H3, and H8 with NA subtypes not determined. Of the 85 positive samples, 50 representing all the subtypes determined by HI/NI assays in this study were subjected to NGS for subtype confirmation and genetic characterisation. We obtained good sequences for 30 samples by NGS. Through NGS, eight HA/NA subtypes (i.e., H1N8, H2N9, H8N4, H10N6, H10N8, H10N9, H11N6, and H11N9) were detected. All the H1N8, H2N9, H8N4, H10N6, H10N9, and H11N9 isolates were detected in 2015 with the majority being samples collected in November. All the two H8N4 and four H11N6 isolates were detected in samples collected in September 2020 and November 2021, respectively. The most prevalent subtypes in the present study were H10N8 (20%; 6/30), followed by H11N9 (16.7%; 5/30). The most prevalent HA subtype was H10 (40%; 12/30) which occurred in combination with three NA (N6, N8, and N9) subtypes.

### 3.2. Nucleotide Sequence Analysis of the Isolates

To further characterise the 30 isolates that were sequenced in this study, the nucleotide similarities of the full-length sequences of all eight segments of AIVs (PB2, PB1, PA, HA, NP, NA, M, and NS) were assessed using BLAST (Table 2). The HA genes of the H1N8, H10N8, H10N9, and H11N9 isolates showed the highest nucleotide sequence similarity (94.4–95.5%) to viruses isolated from ostriches, Pekin ducks, and red-billed teals from South Africa, while the HA genes of the H2N9 and H10N6 isolates shared 95.1% and 94.9% nucleotide sequence identity with A/tufted duck/Georgia/1/2012 (H2N3) and A/duck/Mongolia/371/2010 (H10N8), respectively. The HA genes of the H8N4 and H11N6 isolates showed 97.2–98.9% nucleotide sequence similarity to H8N4 and H11N3 viruses isolated from ducks in Bangladesh in 2019. Analysis of NA gene segments of all isolates indicated that they shared 96.1–98.8% nucleotide sequence identity with viruses isolated in wild waterfowl from Asia, Africa, and Europe (Table 2).

The nucleotide sequence analysis further revealed that the PB2 genes of the H1N8, H2N9, H10N6, H10N8, H10N9, and H11N9 isolates were highly similar (97.9–98.6%) to A/tufted duck/Georgia/1/2012 (H2N3), while those of the H8N4 and H11N6 isolates were similar (97.2–97.4%) to A/pintail/Egypt/MB-D-384C/2015 (H3N6). The PB1 genes of the H1N8 isolates showed high nucleotide similarity (98.2%) to A/shelduck/South Africa/DLH/2012 (H7N8). The PA genes of the HIN8, H2N9, H10N8, and H10N9 isolates showed 98.2–98.5% nucleotide similarity to an H7N7 virus, A/ostrich/South Africa/KRB/2013 (H7N7), while the NP genes of the H10N8, H10N9, and H11N9 isolates showed a high nucleotide identity of 98.5–98.7% with an H7N8 virus isolated in Egypt (Table 2).

### 3.3. Phylogenetic Analysis of the Viral Surface Glycoprotein Genes

**Figure 1:**
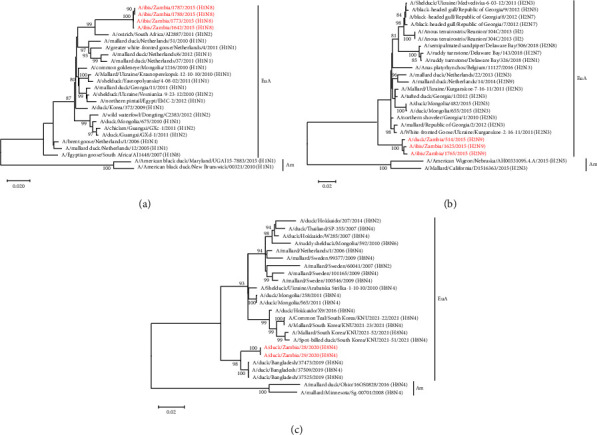
Phylogenetic analysis of the H1, H2, and H8 genes of AIVs: (a) phylogenetic tree of H1 genes based on 1702 nucleotides; (b) phylogenetic tree of H2 genes based on 1695 nucleotides; (c) phylogenetic tree of H1 genes based on 1712 nucleotides. The viruses isolated in this study are in red text. Numbers at branch nodes are bootstrap values ≥ 70%. Am–American lineage; EuA–Eurasian lineage. Bar, number of substitutions per site.

**Figure 2:**
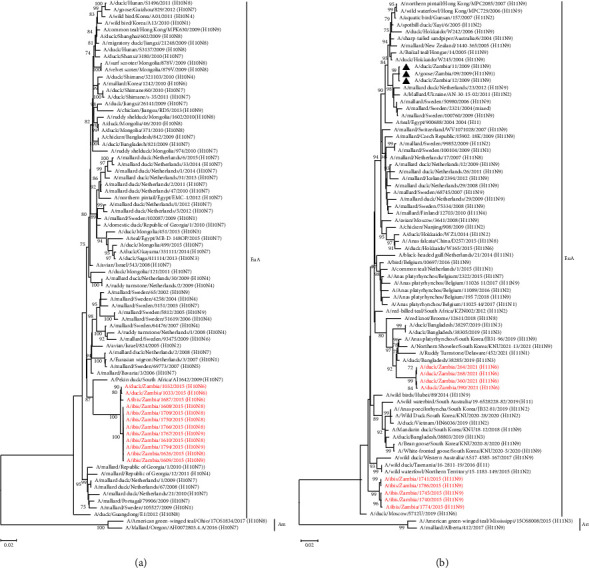
Phylogenetic analysis of the H10 and H11 HA genes of AIVs: (a) phylogenetic tree of H10 gene based on 1686 nucleotides; (b) phylogenetic tree of H11 gene based on 1707 nucleotides. The viruses isolated in this study are in red text, while those previously isolated in Zambia are shown with a black triangle. Numbers at branch nodes are bootstrap values ≥ 70%. Am–American lineage; EuA–Eurasian lineage. Bar, number of substitutions per site.

**Figure 3:**
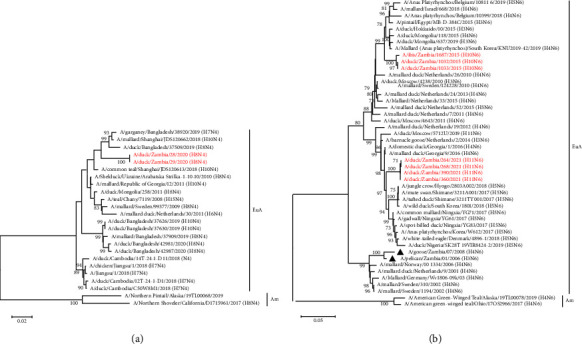
Phylogenetic analysis of the N4 and N6 NA genes: (a) phylogenetic tree of N4 based on 1405 nucleotides; (b) phylogenetic tree of N6 based on 1413 nucleotides. The viruses isolated in this study are in red text, while those previously isolated in Zambia are shown with a black triangle. Numbers at branch nodes are bootstrap values ≥ 70%. Am–American lineage; EuA–Eurasian lineage. Bar, number of substitutions per site.

**Figure 4:**
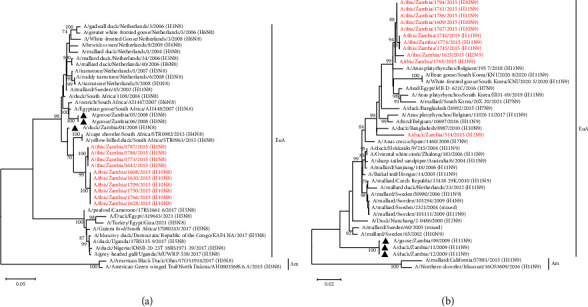
Phylogenetic analysis of the N8 and N9 NA genes. (a) Phylogenetic tree of N8 based on 1413 nucleotides; (b) phylogenetic tree of N9 based on 1413 nucleotides. The viruses isolated in this study are in red text, while those previously isolated in Zambia are shown with a black triangle. Numbers at branch nodes are bootstrap values ≥ 70%. Am–American lineage; EuA–Eurasian lineage. Bar, number of substitutions per site.

Phylogenetic analysis of the HA and NA gene sequences revealed that the viruses isolated in the current study belonged to the Eurasian virus lineage (Figures 1–4). Analysis of the tree topology of the HA gene indicated that the H1N8, H2N9, H8N4, H10N6, H10N8, and H10N9 isolates grouped together and formed distinguishable clusters in their respective trees (Figures 1 and 2). The H11N6 viruses isolated in 2021 clustered distinctly from the H11N9 viruses isolated in 2015. A/ostrich/South Africa/AI2887/2011 (H1N2) formed a precursor-like relationship to the H1N8 isolates characterised in this study (Figure 1(a)). Interestingly, the HA genes of the H2N9 isolates formed a separate and distinct “African lineage-like” cluster and were not closely related to the H2Nx viruses detected in Réunion Island (Figure 1(b)). The analysis further indicated that the HA genes of the H8N4 isolates were closely related to the H8N4 viruses isolated in Bangladesh in 2019 (Figure 1(c)). Similar to the H1 gene tree, the virus A/pekin duck/South Africa/AI1642/2009 (H10N7) had a precursor-like relationship to the HA gene of all the H10 isolates characterised in this study (Figure 2(a)). The H11 HA phylogeny revealed that the H11N6 and H11N9 isolates formed distinct clusters and were distantly related to H11N9 viruses previously isolated in Zambia in 2009 (Figure 2(b)).

Phylogenetic analysis of the NA gene revealed that the H8N4 isolates of the current study formed a separate cluster and were closely related to viruses isolated from wild waterfowl in Asia (Figure 3(a)). The N6 NA genes of the H10N6 and H11N6 viruses isolated in 2015 and 2021 formed distinct clusters based on the year of isolation and were distantly related to the NA genes of the H3N6 and H4N6 viruses isolated in Zambia previously (Figure 3(b)). Analysis of the NA genes of the H1N8 and H10N8 isolates indicated that all the isolates grouped together and were closely related to viruses isolated from wild waterfowl in South Africa (A/cape shoveler/South Africa/STR0982/2013 (H4N8); A/yellow-billed duck/South Africa/STR0963/2013 (H4N8)) and Zambia (A/duck/Zambia/04/2008 (H3N8)) (Figure 4(a)). Except for A/duck/Zambia/514/2015 (H2N9) which clustered separately, the N9 NA gene sequences of the other N9 viruses isolated in the current study were closely related to each other and belonged to a clade that included viruses from Belgium, South Korea, and Egypt (Figure 4(b)).

### 3.4. Phylogenetic Analysis of the Viral Internal Protein Genes

**Figure 5:**
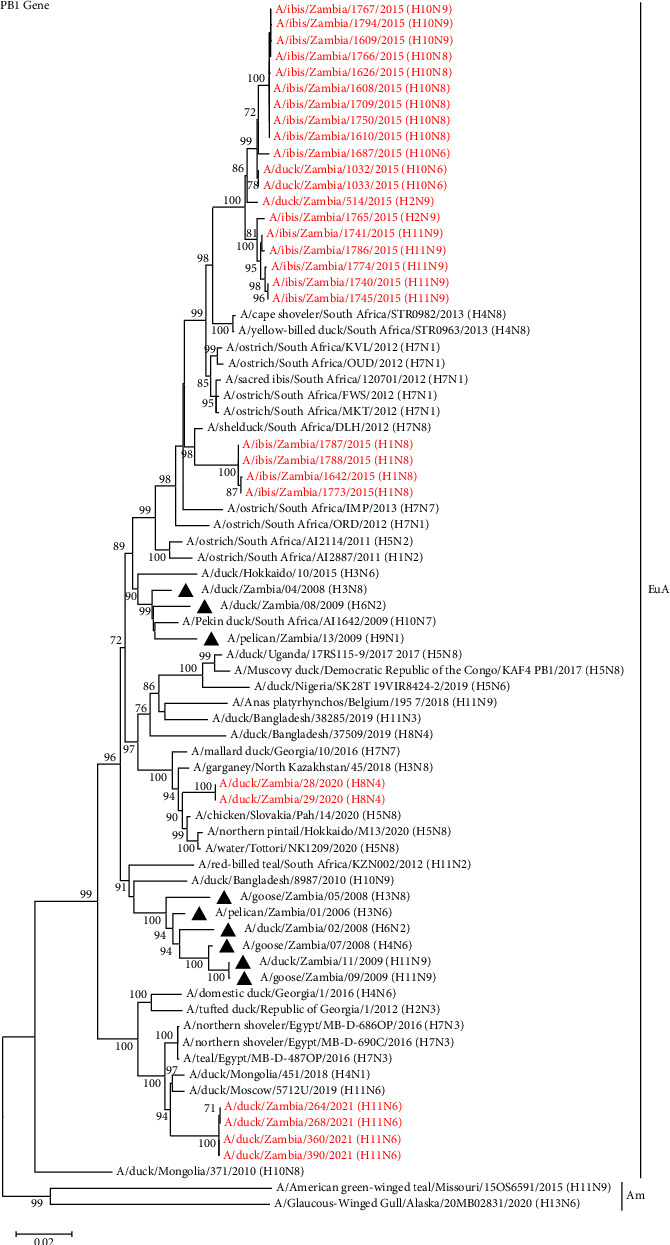
Phylogenetic analysis of the PB1 genes based on 2279 nucleotides. The viruses isolated in this study are in red text, while those previously isolated in Zambia are shown with a black triangle. Numbers at branch nodes are bootstrap values ≥ 70%. Am–American lineage; EuA–Eurasian lineage. Bar, number of substitutions per site.

**Figure 6:**
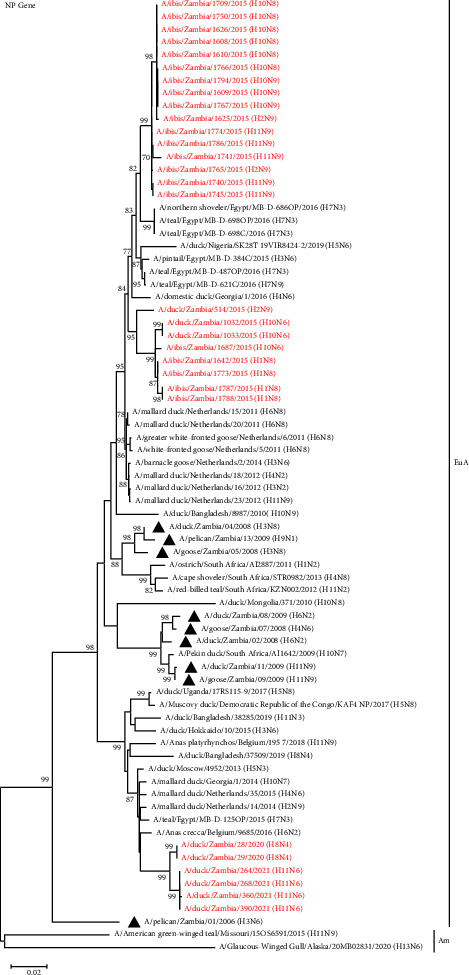
Phylogenetic analysis of the NP genes based on 1506 nucleotides. The viruses isolated in this study are in red text, while those previously isolated in Zambia are shown with a black triangle. Numbers at branch nodes are bootstrap values ≥ 70%. Am–American lineage; EuA–Eurasian lineage. Bar, number of substitutions per site.

**Figure 7:**
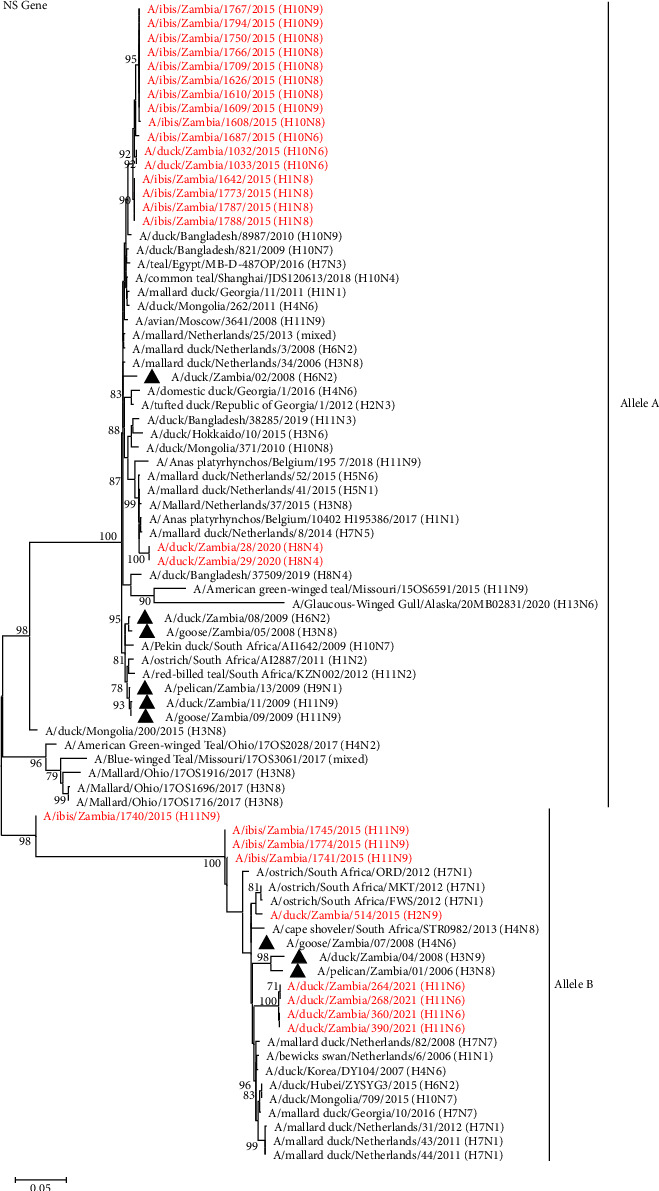
Phylogenetic analysis of the AIV NS genes based on 852 nucleotides. The viruses isolated in this study are in red text, while those previously isolated in Zambia are shown with a black triangle. Numbers at branch nodes are bootstrap values ≥ 70%. Bar, number of substitutions per site.

In general, the tree topologies of all internal protein genes revealed that the viruses isolated in the present study clustered among the Eurasian virus lineage (Figures 5–7, Supplemental Figures S1–S3). The analysis also revealed that the viruses isolated in 2015 formed separate clusters from viruses isolated in 2020 and 2021 and were distinct from those previously analysed in 2008–2009, except for the M and NS gene segments, whose sequences were clustered with some of the Zambian viral sequences characterised previously (Figures 5–7, Supplemental Figures S1–S3). Some internal protein genes characterised in this study were closely related to H7 LPAIVs.

The PB1 genes of the viruses isolated in 2015 were closely related to A/yellow-billed duck/South Africa/STR0963/2013 (H4N8) and A/cape shoveler/South Africa/STR0982/2013 (H4N8) except for PB1 genes of the H1N8 isolates which had a close relationship to an H7N8 LPAIV isolate, A/shelduck/South Africa/DLH/2012 (H7N8), as shown in Figure 5. Additionally, the PB1 genes of the H8N4 isolates were closely related to that of viruses isolated in Japan and Slovakia, while those of the H11N6 isolates were closely related to viruses isolated in Moscow (A/duck/Moscow/5712U/2019 (H11N6)) and Mongolia (A/duck/Mongolia/451/2018 (H4N1)) (Figure 5).

The NP gene phylogeny revealed that the viruses isolated in 2020–2021 formed a single cluster related to viruses isolated in Belgium, Netherlands, Georgia, Russia, and Egypt (Figure 6). In contrast, viruses isolated in 2015 formed two clusters, with the majority of the sequences belonging to a clade that included H7N3 viruses detected from wild birds in Egypt in 2016 (Figure 6).

Phylogenetic analysis of the NS gene demonstrated that the NS genes of the isolates characterised in the current study grouped into two separate alleles, alleles A and B, with the majority of the viruses being grouped into allele A (Figure 7). The H11N6 isolates in allele B formed a distinct cluster and clustered closely with viruses isolated in Zambia in 2006 and 2008 and those isolated in Europe and Asia. In addition, the Zambian allele B sequences clustered close to H7N1 South African viruses detected in ostriches in 2012 (Figure 7). Within allele A, all the NS gene sequences of the H1 and H10 isolates were closely related to A/duck/Bangladesh/8987/2010 (H10N9) (Figure 7). In contrast, the H8N4 isolates belonged to a cluster of isolates detected from wild birds mainly in the Netherlands and included H5N1, H5N6, and H7N5 LPAIVs, though none of these was closely related to our isolates (Figure 7).

### 3.5. Genetic Analysis of the HA Cleavage Site

Genetic analysis of the HA gene segment indicated that the isolates with the same HA subtypes shared similar motifs at the cleavage site of the HA protein as follows: H1N8 (PSIQSR/GLF), H2N9 (PQIESR/GLF), H8N4 (PSIEPK/GLF), H10N6/H10N8/H10N9 (PEVMQGR/GLF), and H11N6/H11N9 (PAIASR/GLF). The sequences for all the isolates were typical for LPAIVs as none of the viruses had multiple basic amino acids at the HA cleavage site.

## 4. Discussion

In this study, we found evidence of AIV circulation in the wild waterfowl found in LNP in Zambia with a positivity rate of 3.0% during the three years of sampling with the highest positivity rate being detected in 2015 (4.1%). The positivity rate of AIVs in wild waterfowl in this study was consistent with the finding of the previous review which found a prevalence of 3% in birds in Africa [48]. While bird species in the order *Anseriformes* and *Charadriiformes* are known to be the main natural reservoir of AIV, the highest positivity rate in the current study was detected in glossy ibis (*Plegadis falcinellus*), order *Pelecaniformes*. This is the first study to report AIVs in glossy ibis in Zambia. AIV has also been detected in African sacred ibis (*Threskiornis aethiopicus*) in South Africa [49], and the first AIV detected in Zambia was from a great white pelican [37] which is also from the order *Pelecaniformes*. These data suggest that birds of the order *Pelecaniformes* may be frequently infected with AIVs and may play an important role in the eco-epidemiology of these viruses. Moreover, experimental infection of adult ibis has revealed their susceptibility to and capability of shedding multiple AIV subtypes [50].

In this study, we found that the isolation rate of AIVs was higher in the wet season compared to the dry season. This could be attributed to the presence of migratory waterfowl which are known to be natural reservoirs of AIVs as most of the migratory birds begin to arrive in LNP from November to April, which coincides with the wet season in Zambia. Further, our findings corroborate the general understanding that the prevalence of AIVs tends to increase during the period when Eurasian migratory water birds overwinter in sub-Saharan Africa and decrease after they migrate back to Eurasia [51]. However, our findings do not agree with the previous review report that found a higher prevalence in the dry season in sub-Saharan Africa [48]. Higher prevalence in dry seasons could be attributed to limited water bodies that may allow increased interaction of waterfowl by congregating at particular sites, which provides opportunities for AIV transmission as well as detection during surveillance activities [48]. The difference in the findings could be because sampling was not carried out in some months of 2020–2021 due to restrictions of movements brought about by the COVID-19 pandemic. Despite a higher positivity rate of AIV in the wet season, the isolation of AIVs in the dry season in this study may denote that AIV transmission by wild birds may be possible at any time of the year.

In the current study, we detected eight LPAIV subtypes. Although there was a disparity in the HA/NA subtypes obtained using HI/NI assays and NGS, the detected subtypes included H1N8, H2N9, H8N4, H10N6, H10N8, H10N9, H11N6, and H11N9. The disparity could be due to possible cross-reactivity of some AIV subtypes [43, 52], a pitfall that was resolved by NGS.

Along with previous studies [36, 37], the total number of HA and NA subtypes that have been identified in Zambia are nine (H1–H4, H6, and H8–H11) and six NA (N1, N2, N4, N6, N8, and N9), respectively. These findings demonstrated a considerably high HA and NA diversity of AIVs circulating among wild waterfowl in Zambia. Previous studies conducted in Zambia have also reported the isolation of LPAIVs from ducks, geese, and pelicans [36, 37] within LNP, indicating the continuous circulation of these viruses among migratory and indigenous bird species in the park. The findings confirm the idea that wild waterfowl are important in the maintenance and introduction of a wide range of viruses into the Zambian environment. Similar studies in Africa have also reported LPAIVs in different avian species including wild and domestic birds [30, 32, 34, 53, 54]. However, to the best of our knowledge, no H2N9, H8N4, and H10N8 viruses have been reported in Africa, and we did not find any sequences of these subtypes on the continent in GenBank or GISAID (i.e., the Global initiative on sharing all influenza data). This suggests that this is the first time that these HA/NA combinations are being reported in Zambia and Africa as a whole. For some subtypes that we are reporting now such as H10N6, H10N9, and H11N6, though they have been reported previously [32, 55, 56], sequence data are not available, and therefore, this study adds to the genetic resource of AIV detected in Africa. Hence, continuous surveillance and monitoring of wild waterfowl for AIVs should be supported to facilitate the creation of a library of isolates circulating in Africa that can be used for diagnosis and control strategies such as vaccine development in the event that any of these viruses causes an outbreak in poultry or other mammals including humans.

Phylogenetic analysis of all eight gene segments of AIVs revealed that the viruses isolated in the current study clustered with the viruses of the Eurasian lineage. However, it was noted that the HA genes of H2N9 viruses clustered separately from the major Eurasian clade, which may suggest possible independent evolution of these genes and raises the temptation to speculate on the possible existence of an African lineage of AIVs. The analysis further revealed that AIVs characterised in this study and those previously isolated in Zambia grouped into distinct clusters according to the period of isolation, signifying that these viruses were introduced in the Zambian environment independently at different times. Most of the genes were closely related to AIVs isolated from wild and domestic birds in Bangladesh, Belgium, Egypt, Georgia, Mongolia, the Netherlands, and South Africa. The close phylogenetic clustering of sequences analysed in this study with those of Eurasian isolates, along with the observation that most sequences characterised herein were distantly related to those previously isolated in Zambia, may suggest that these viruses were introduced in the country by Palearctic migratory birds. Additionally, the viral internal protein genes of some viruses in the current study were closely related to notifiable H7 LPAIVs, indicating possible gene exchange with viruses with the potential to mutate into HPAIVs. Remarkably, only the PB2 and NS genes of the H8N4 and H11N6 viruses isolated in 2020 and 2021 were closely related to AIVs isolated in Africa. Therefore, this might indicate a gap in the surveillance of AIVs in Africa or that these viruses were recently introduced into the African ecosystem. Furthermore, our phylogenetic analysis showed that some of the AIV genes studied were closely related to those identified in poultry, confirming the understanding that the wild waterfowl population act as a source of AIV infection for domestic birds. The findings highlight the need for continuous surveillance of AIVs in both wild and domestic birds to monitor the introduction of viruses of veterinary and public health significance.

This study was not without limitations. We did not carry out sampling in all the months of the dry season in the 2020 and 2021 sampling periods due to the emergence of the COVID-19 pandemic and the associated public health measures that involved movement restrictions. In addition, no sampling was carried out during the 2016 to 2019 period which leaves a considerable gap in our understanding of AIV ecology and epidemiology in the country and region at large. Furthermore, we did not use molecular tools in wild waterfowl species identification but identified them morphologically. The inclusion of an ornithologist from the Department of National Parks and Wildlife alleviated this shortcoming.

## 5. Conclusion

Our study revealed the active circulation of multiple LPAIV subtypes in wild waterfowl in Zambia and reports for the first time the isolation of AIVs in glossy ibis in Africa. Phylogenetic analysis revealed that the AIVs isolated in this study clustered with isolates of the Eurasian lineage. The viruses isolated in 2015 formed separate clusters from those isolated in 2020–2021 suggesting independent introductions of AIVs in wild waterfowl in Zambia. Further, some internal protein genes clustered with H7 LPAIVs isolated from South Africa and Egypt. This study emphasises the importance of continuous surveillance and monitoring of AIVs in wild waterfowl including birds traditionally not considered to be major reservoirs of AIVs in order to be better prepared to protect animal and public health from zoonotic influenza.

## Figures and Tables

**Table 1:** Number of samples collected and positivity rates over the surveillance period 2015, 2020, and 2021.

Variable	No. of samples collected	No. of AIV positive samples (%)	AIV subtypes detected
Sample Type			
Faecal	2851	85 (3.0)	H1N8, H2N9, H8N4, H10N6, H10N8, H10N9, H11N6, and H11N9
Bird species			
Ducks	1214	10 (0.8)	H2N9, H8N4, H10N6, and H11N6
Geese	1227	3 (0.2)	H10N8 and H11N9
Ibises	302	72 (23.8)	H1N8, H2N9, H10N6, H10N8, and H11N9
Pelicans	98	0 (0)	—
White egrets	10	0 (0)	—
Season			
Dry†	1539	7 (0.5)	H2N9, H8N4, H10N6, and H11N9
Wet ‡	1312	78 (5.9)	H1N8, H2N9, H10N6, H10N8, H10N9, H11N6, and H11N9
Sampling year			
2015	1921	79 (4.1)	H1N8, H2N9, H10N6, H10N8, H10N9, and H11N9
2020	242	2 (0.8)	H8N4
2021	688	4 (0.6)	H11N6

†Dry season: May–October; ‡wet season: November–April.

**Table 2:** Influenza viruses with the highest nucleotide sequence similarity to viruses in the current study.

Representative virus subtype	Gene segment	Highest homology influenza A virus	GenBank accession #	% homology
H1N8	PB2	A/tufted duck/Georgia/1/2012 (H2N3)	MF147767.1	97.9
	PB1	A/shelduck/South Africa/DLH/2012 (H7N8)	KT777839.1	98.2
	PA	A/ostrich/South Africa/KRB/2013 (H7N7)	KT777875.1	98.2
	HA	A/ostrich/South Africa/AI2887/2011 (H1N2)	JX069105.1	95.5
	NP	A/mallard duck/Netherlands/18/2012 (H4N2)	MF146131.1	97.8
	NA	A/duck/Zambia/04/2008 (H3N8)	AB569497.1	96.5
	M	A/Mallard/Netherlands/31/2014 (H4N3)	MK414709.1	98.3
	NS	A/mallard/Netherlands/25/2013 (mixed)	MK192309.1	99.0

H2N9	PB2	A/tufted duck/Georgia/1/2012 (H2N3)	MF147767.1	98.5
	PB1	A/yellow-billed duck/South Africa/STR0963/2013 (H4N8)	KT777923.1	97.7
	PA	A/ostrich/South Africa/KRB/2013 (H7N7)	KT777875.1	98.5
	HA	A/tufted duck/Georgia/1/2012 (H2N3)	MF146097.1	95.1
	NP	A/mallard duck/Netherlands/15/2011 (H6N8)	KX979542.1	98.5
	NA	A/duck/Bangladesh/8987/2010 (H10N9)	MH071484.1	96.7
	M	A/*Anas platyrhynchos*/Belgium/108116/2019 (H5N6)	MT406810.1	98.2
	NS	A/ostrich/South Africa/MKT/2012 (H7N1)	KT777895.1	99.4

H8N4	PB2	A/pintail/Egypt/MB-D-384C/2015 (H3N6)	MN208007.1	97.4
	PB1	A/garganey/North Kazakhstan/45/2018 (H3N8)	MT126633.1	98.4
	PA	A/duck/Mongolia/451/2018 (H4N1)	MW188636.1	97.7
	HA	A/duck/Bangladesh/37509/2019 (H8N4)	MT090424.1	98.0
	NP	A/duck/Moscow/4952/2013 (H5N3)	MN588198.1	97.6
	NA	A/common teal/Shanghai/JDS120613/2018 (H10N4)	MN049535.1	96.2
	M	A/duck/Mongolia/451/2018 (H4N1)	MW188640.1	98.9
	NS	A/mallard duck/Netherlands/41/2015 (H5N1)	MF694125.1	99.1

H10N6	PB2	A/tufted duck/Georgia/1/2012 (H2N3)	MF147767.1	97.9
	PB1	A/yellow-billed duck/South Africa/STR0963/2013 (H4N8)	KT777923.1	97.7
	PA	A/cape shoveler/South Africa/STR0982/2013 (H4N8)	KT777929.1	97.8
	HA	A/Pekin duck/South Africa/AI1642/2009 (H10N7)	GQ404728.2	95.0
	NP	A/mallard duck/Netherlands/18/2012 (H4N2)	MF146131.1	97.8
	NA	A/duck/Hokkaido/10/2015 (H3N6)	LC339733.1	97.0
	M	A/Mallard/Netherlands/31/2014 (H4N3)	MK414709.1	98.3
	NS	A/duck/Bangladesh/821/2009 (H10N7)	MH071464.1	99.0

H10N8/H10N9	PB2	A/tufted duck/Georgia/1/2012 (H2N3)	MF147767.1	98.5
	PB1	A/yellow-billed duck/South Africa/STR0963/2013 (H4N8)	KT777923.1	97.3
	PA	A/ostrich/South Africa/KRB/2013 (H7N7)	KT777875.1	98.3
	HA	A/Pekin duck/South Africa/AI1642/2009 (H10N7)	GQ404728.2	94.8
	NP	A/teal/Egypt/MB-D-487OP/2016(H7N3)	MN208011.1	98.5
	NA	A/cape shoveler/South Africa/STR0982/2013 (H4N8)	KT777932.1	96.1
	M	A/teal/Egypt/MB-D-698OP/2016 (H7N3)	MN207981.1	99.5
	NS	A/duck/Bangladesh/821/2009 (H10N7)	MH071464.1	98.8

H11N6	PB2	A/pintail/Egypt/MB-D-384C/2015 (H3N6)	MN208007.1	97.2
	PB1	A/duck/Mongolia/451/2018 (H4N1)	MW188635.1	97.8
	PA	A/duck/Mongolia/451/2018 (H4N1)	MW188636.1	97.5
	HA	A/duck/Bangladesh/38285/2019 (H11N3)	MT090343.1	97.2
	NP	A/mallard duck/Netherlands/35/2015 (H4N6)	MF694210.1	97.5
	NA	A/domestic duck/Georgia/1/2016 (H4N6)	MF694247.1	97.4
	M	A/duck/Mongolia/451/2018 (H4N1)	MW188640.1	98.9
	NS	A/ostrich/South Africa/MKT/2012 (H7N1)	KT777895.1	97.9

H11N9	PB2	A/tufted duck/Georgia/1/2012 (H2N3)	MF147767.1	98.3
	PB1	A/yellow-billed duck/South Africa/STR0963/2013 (H4N8)	KT777923.1	97.5
	PA	A/cape shoveler/South Africa/STR0982/2013 (H4N8)	KT777929.1	97.8
	HA	A/red-billed teal/South Africa/KZN002/2012 (H11N2)	KT777885.1	97.4
	NP	A/teal/Egypt/MB-D-487OP/2016 (H7N3)	MN208011.1	98.7
	NA	A/Anas platyrhynchos/Belgium/195_7/2018 (H11N9)	MT406955.1	98.6
	M	A/*Anas platyrhynchos*/Belgium/10811_6/2019(H5N6)	MT406810.1	98.5
	NS	A/mallard duck/Netherlands/43/2011 (H7N1)	KX979531.1	97.3

## Data Availability

All data generated in this study are included within the article along with their supplementary files.

## Supplementary Materials

Supplementary Materials Figure S1. Phylogenetic analysis of the AIV PB2 genes based on 2280 nucleotides. The viruses isolated in this study are in red text, while those previously isolated in Zambia are shown with a black triangle. Numbers at branch nodes are bootstrap values ≥ 70%. Am–American lineage; EuA–Eurasian lineage. Bar, number of substitutions per site. Figure S2. Phylogenetic analysis of the AIV PA genes based on 2118 nucleotides. The viruses isolated in this study are in red text, while those previously isolated in Zambia are shown with a black triangle. Numbers at branch nodes are bootstrap values ≥ 70%. Am–American lineage; EuA–Eurasian lineage. Bar, number of substitutions per site. Figure S3. Phylogenetic analysis of the AIV M genes based on 989 nucleotides. The viruses isolated in this study are in red text, while those previously isolated in Zambia are shown with a black triangle. Numbers at branch nodes are bootstrap values ≥ 70%. Am–American lineage; EuA–Eurasian lineage. Bar, number of substitutions per site.
