# Supplementary figures and images for: Surveillance and Phylogenetic Characterisation of Avian Influenza Viruses Isolated from Wild Waterfowl in Zambia in 2015, 2020, and 2021
Source: Transbound Emerg Dis. 2023 Mar 1;2023:4606850. doi: 10.1155/2023/4606850 (PMC12016966; doi:10.1155/2023/4606850)

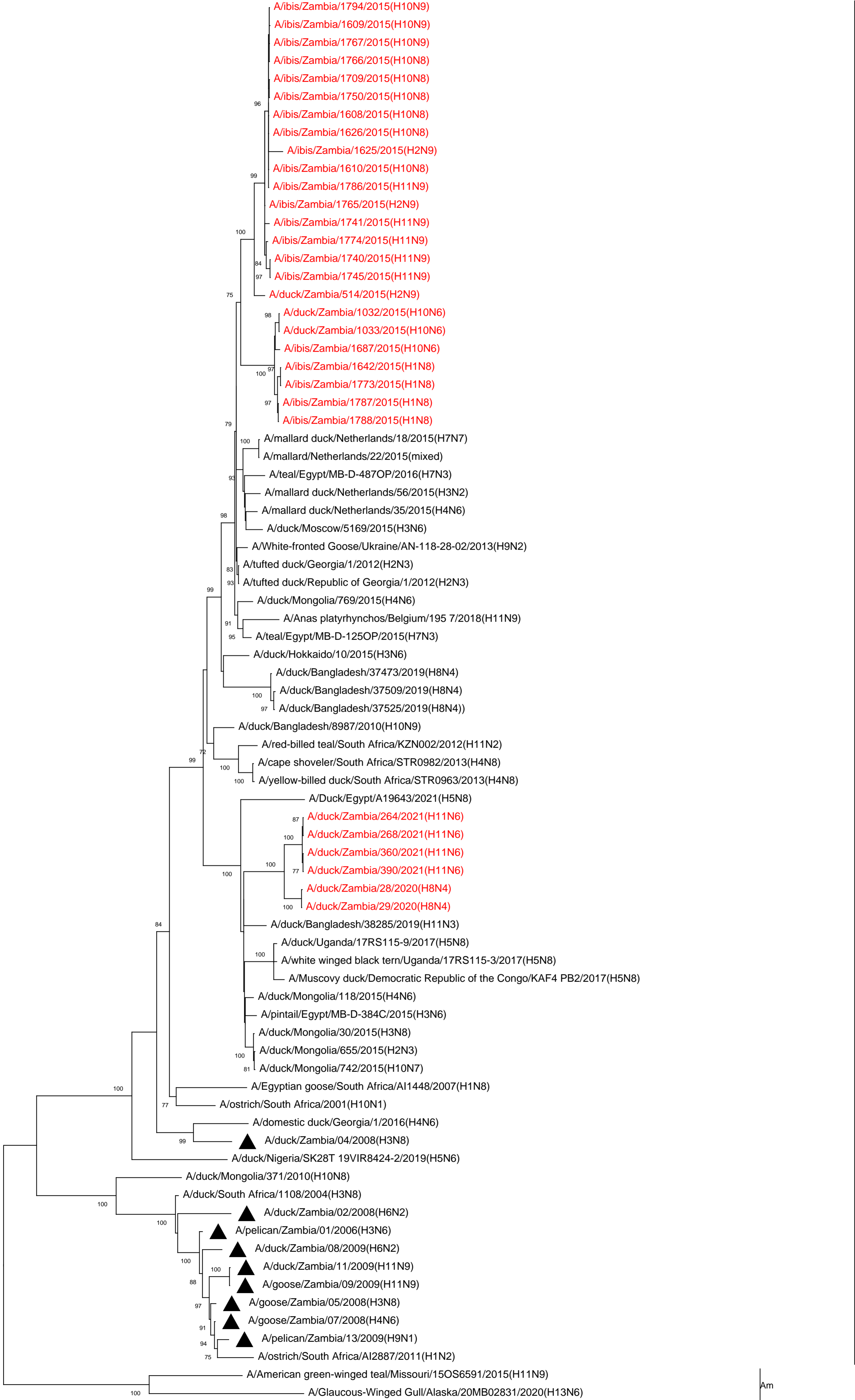

0.02

Am

Supplement: Supplementary Materials — Figure S1. Phylogenetic analysis of the AIV PB2 genes based on 2280 nucleotides. The viruses isolated in this study are in red text, while those previously isolated in Zambia are shown with a black triangle. Numbers at branch nodes are bootstrap values ≥ 70%. Am–American lineage; EuA–Eurasian lineage. Bar, number of substitutions per site. Figure S2. Phylogenetic analysis of the AIV PA genes based on 2118 nucleotides. The viruses isolated in this study are in red text, while those previously isolated in Zambia are shown with a black triangle. Numbers at branch nodes are bootstrap values ≥ 70%. Am–American lineage; EuA–Eurasian lineage. Bar, number of substitutions per site. Figure S3. Phylogenetic analysis of the AIV M genes based on 989 nucleotides. The viruses isolated in this study are in red text, while those previously isolated in Zambia are shown with a black triangle. Numbers at branch nodes are bootstrap values ≥ 70%. Am–American lineage; EuA–Eurasian lineage. Bar, number of substitutions per site. [file 4606850.f1.zip › Figure S1_PB2 Gene.pdf]

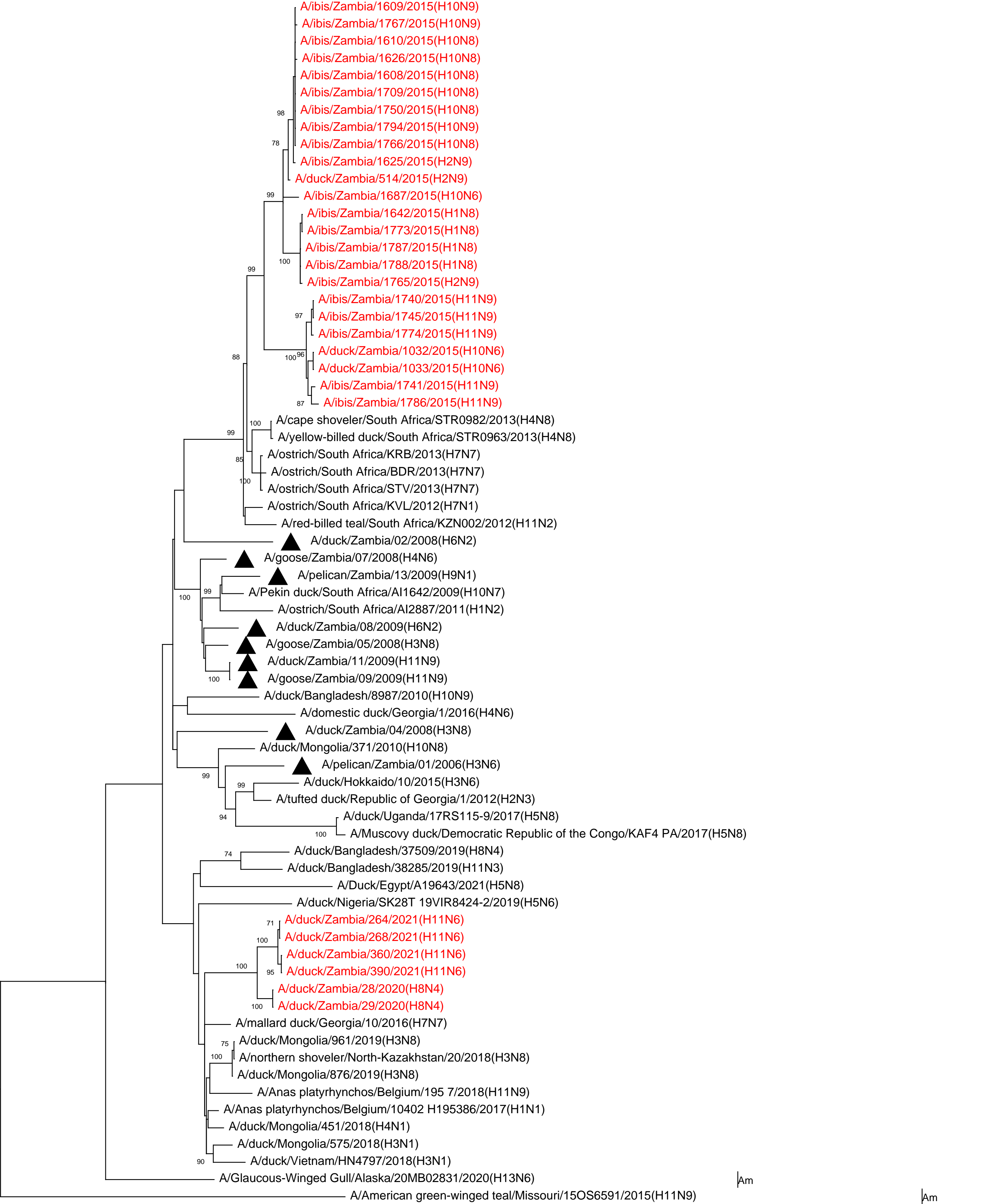

0.02

Am

Am

EuA

Supplement: Supplementary Materials — Figure S1. Phylogenetic analysis of the AIV PB2 genes based on 2280 nucleotides. The viruses isolated in this study are in red text, while those previously isolated in Zambia are shown with a black triangle. Numbers at branch nodes are bootstrap values ≥ 70%. Am–American lineage; EuA–Eurasian lineage. Bar, number of substitutions per site. Figure S2. Phylogenetic analysis of the AIV PA genes based on 2118 nucleotides. The viruses isolated in this study are in red text, while those previously isolated in Zambia are shown with a black triangle. Numbers at branch nodes are bootstrap values ≥ 70%. Am–American lineage; EuA–Eurasian lineage. Bar, number of substitutions per site. Figure S3. Phylogenetic analysis of the AIV M genes based on 989 nucleotides. The viruses isolated in this study are in red text, while those previously isolated in Zambia are shown with a black triangle. Numbers at branch nodes are bootstrap values ≥ 70%. Am–American lineage; EuA–Eurasian lineage. Bar, number of substitutions per site. [file 4606850.f1.zip › Figure S2_PA Gene.pdf]

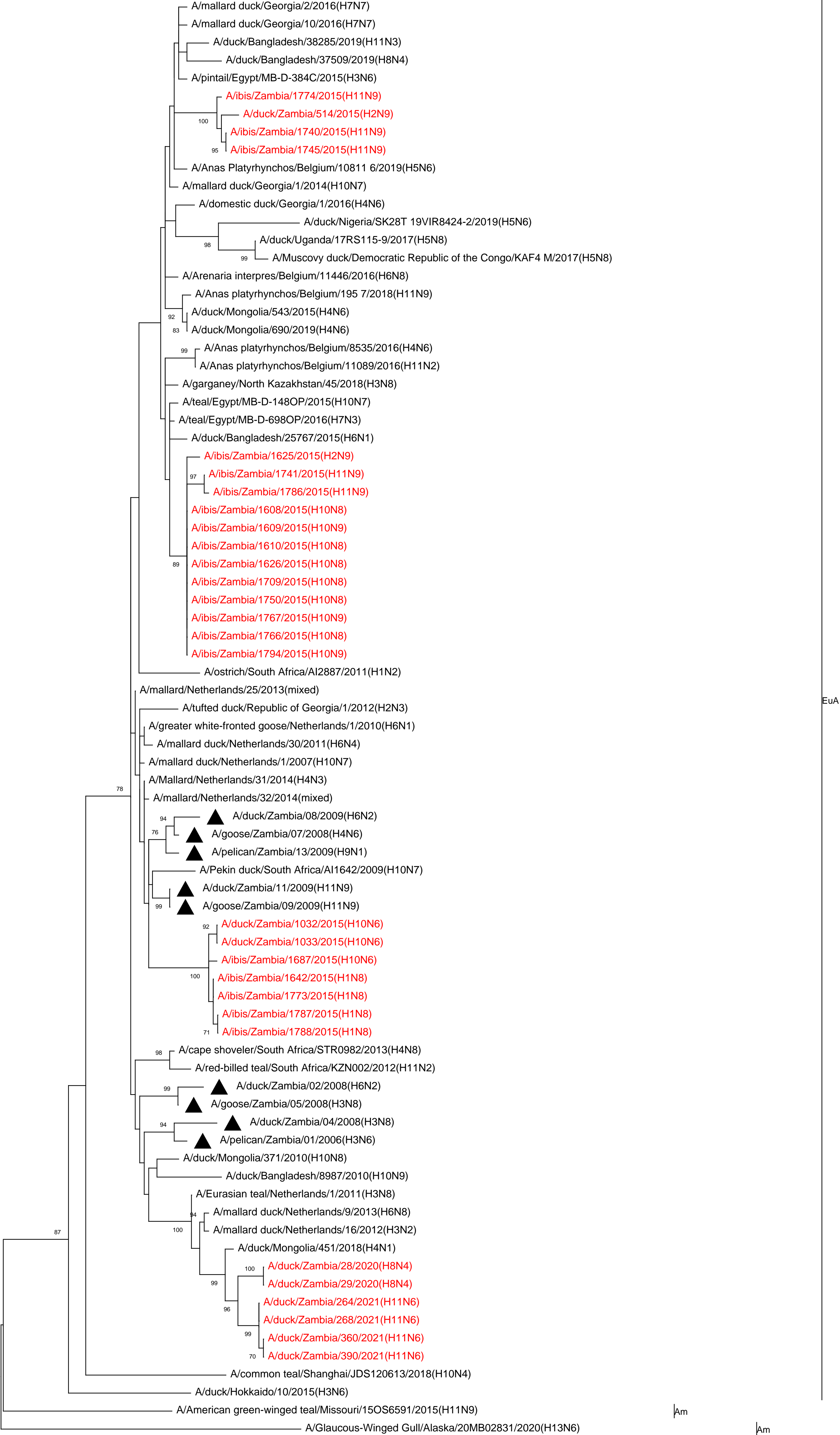

0.01

Am

Am

Supplement: Supplementary Materials — Figure S1. Phylogenetic analysis of the AIV PB2 genes based on 2280 nucleotides. The viruses isolated in this study are in red text, while those previously isolated in Zambia are shown with a black triangle. Numbers at branch nodes are bootstrap values ≥ 70%. Am–American lineage; EuA–Eurasian lineage. Bar, number of substitutions per site. Figure S2. Phylogenetic analysis of the AIV PA genes based on 2118 nucleotides. The viruses isolated in this study are in red text, while those previously isolated in Zambia are shown with a black triangle. Numbers at branch nodes are bootstrap values ≥ 70%. Am–American lineage; EuA–Eurasian lineage. Bar, number of substitutions per site. Figure S3. Phylogenetic analysis of the AIV M genes based on 989 nucleotides. The viruses isolated in this study are in red text, while those previously isolated in Zambia are shown with a black triangle. Numbers at branch nodes are bootstrap values ≥ 70%. Am–American lineage; EuA–Eurasian lineage. Bar, number of substitutions per site. [file 4606850.f1.zip › Figure S3_M Gene.pdf]
